# Combining a national research autopsy programme with computational approaches to study cancer evolution and metastasis: an interview with Mariam Jamal-Hanjani

**DOI:** 10.1038/s42003-022-03550-2

**Published:** 2022-06-28

## Abstract

Mariam Jamal-Hanjani is the Senior Clinical Lecturer & Group Leader for the Cancer Metastasis Lab at the UCL Cancer Institute and Honorary Consultant in Translational Lung Oncology at UCL Hospital. Mariam is the lead for their PEACE (Posthumous Evaluation of Advanced Cancer Environment) study, which involves people living with incurable cancer donating their bodies for research after they die, so that scientists can learn more about why cancer spreads and how advanced cancer kills.

**Figure:**
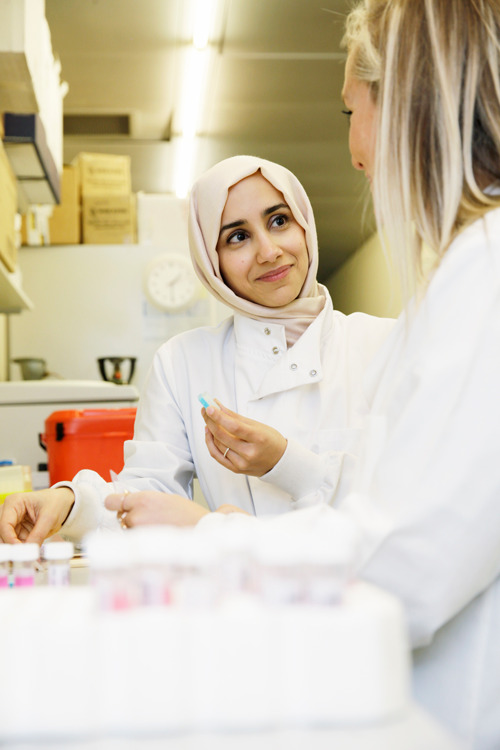
Cancer Research UK

Tell us about your research interests!

My lab is interested in understanding the biological processes that facilitate metastatic cancer and the features of cancer cells allow them to disseminate from the primary tumour to distant metastatic sites. Metastatic disease is the cause of almost 90% of cancer-related deaths, and therefore there is a great need to understand the mechanisms adopted by cancer cells in metastatic seeding. If we can better understand these mechanisms, we may be able to identify features and traits of primary tumours that dictate whether metastasis will occur.

One particular area we are interested in is how cancer cells can alter their metabolism and interact with their surrounding environment to aid the development of metastasis, often resulting in a catabolic state such that patients develop cachexia where muscle and/or fat loss, as well as weight loss, occurs. Cachexia poses a significant unmet need in patients with cancer, and is associated with frailty, intolerance of cancer therapies and poor prognosis. We are investigating this in lung cancer within the national TRACERx study, led by Charles Swanton, by analysing changes in body composition in patients from early to late stage disease using longitudinal imaging, to identify those who develop the cachexia phenotype, and then integrating this with both tumour-intrinsic and extrinsic features, to shed light on the underlying biological mediators.

You obtained your degrees in physics and medicine. What made you move into cancer research and do you think having an interdisciplinary background helped with this?

I have always enjoyed mathematics (the language with which we can describe the universe) and physics (the poetry of this language), and so it felt very natural for me to study physics at university, as I was enamoured by the beauty of quantum physics—a wonderful mix of probability and philosophy. However, I also was fascinated by human physiology and the mechanics of the human body, and so despite the personal fulfilment I gained from physics, I always felt I needed to do something that was more tangible and would bring me closer to people, hence my motivation to study medicine as a graduate student. I enjoyed medical school and the clinical training that followed, and I purposefully chose to specialise in medical oncology in later years knowing that it would give me the opportunity to do a PhD in cancer research. My time in the lab made it very clear to me that I wanted to combine my clinical training with research so that I could continue to work with, and learn from, scientists and clinicians who were incredibly driven to make the discoveries that would hopefully benefit the very patients I cared for.

My background in physics may have fostered a conceptual way of thinking, but more than anything it’s my clinical work that informs and guides the work I do in the lab.

What research achievement are you most proud of?

Almost a decade ago, my colleague, mentor and friend, Charles Swanton, had the idea of setting up a post-mortem study to access cancer tissue, in particular metastatic tumours, from sites of disease that would otherwise not be possible, to enable us to study cancer evolution and the development of drug resistance. It took the two of us quite a few years, but with the support of our colleagues and the patients and relatives who contribute to the study, we have established a national research autopsy programme called PEACE, which facilitates collaborative cancer research nationally and internationally. This has always felt like an achievement given the obvious sensitivities and logistical complexities entailed, and we have been humbled by the sheer selflessness of our patients and their motivation to improve the outcomes for future cancer patients.

How do you think multi-omics and computational approaches have changed the field of cancer evolution, and what do you think is the most exciting application of these tools?

Both the TRACERx and PEACE studies involve computational analysis of large genomic data sets, and so the use of robust and refined bioinformatic methodologies has been crucial for our understanding of how cancers evolve throughout the course of disease and in metastasis. Given the multi-region tumour sampling we adopt, one limitation with previous computational pipelines is the ability to analyse multiples samples from primary and metastatic tumours in each patient, so that we can more accurately capture spatial heterogeneity in tumours and make inferences regarding cancer evolution. Great efforts have been made by many groups to develop tools and approaches for analysing genomic data, which has also allowed us to explore cancer evolution at the mutational and copy number level with greater resolution, and to reconstruct the evolutionary history of each cancer in each patient.

It’s increasingly expected of us to not focus on one ‘omic’ entity and rightly so—we know that cancer evolution is driven by tumour-intrinsic and -extrinsic factors, and we need to study the disease not only at the level of the tumour but the environment in which it exists—locally and at the level of the whole body. Using orthogonal approaches and a multi-omic analysis to study cancer will enable us to study the disease more holistically, incorporating genomics, transcriptomic, proteomics, metabolomics and so on.

How important is diversity to you and what are the impediments for creating inclusive, equitable research labs and practices?

Diversity in research to me means working with clinicians and scientists with diverse backgrounds—both scientifically and personally. I think it’s important since it can inculcate a diversity in our approach to cancer research and an open mind in our scientific thinking. I think the impediments will sometimes be unique to the experience of the researchers. In my opinion, creating an inclusive and diverse research environment relies on fair and transparent recruitment, funding opportunities that support researchers at different career stages, childcare support for working parents and a strong collaborative and team science ethos. Much of this has to be established at an institutional level, but also within individual labs and collaborating teams.

What would be your advice for women who are considering a career in cancer research?

I would encourage any woman who has the desire and motivation to work in cancer research to be ambitious, take every positive opportunity to grow in experience and knowledge, to build strong working relationships with people you trust and whom you respect, and to use every setback as an opportunity to build resilience. Lean on friends and colleagues when you need to, build a network of support around you and identify the role models and mentors who will guide you. And finally, expect tough times and plan ahead for them, but don’t ever forget to enjoy the science and to always have the patient at the heart of your research.

*This interview was conducted by Associate Editor Eve Rogers*.

